# Dataset of geomagnetic absolute measurements performed by Declination and Inclination Magnetometer (DIM) and nuclear magnetometer during the joint Croatian-Hungarian repeat station campaign in Adriatic region

**DOI:** 10.1016/j.dib.2024.110276

**Published:** 2024-03-04

**Authors:** András Attila Csontos, Danijel Šugar

**Affiliations:** a HUN-REN Institute of Earth Physics and Space Science, Csatkai E. u. 6-8, Sopron H-9400, Hungary; b University of Zagreb, Faculty of Geodesy, Institute for Geomatics, Savska cesta 144A, Zagreb HR-10000, Croatia

**Keywords:** Secular variation of geomagnetic field, Data processing, Fieldwork measurement practice, Technical requirements

## Abstract

The highest level of the present-day geomagnetic recordings is presented by the absolute controlled magnetic measurements. This quality is permanently fulfilled in geomagnetic observatories (GO) only. The absolute records are based on nuclear magnetometers for the intensity measurement and on DIM (Declination and Inclination Magnetometer) instruments for the direction determination of the ambient magnetic field. Although the density of GOs is not dense enough for every scientific purpose (i.e., modelling the secular variation in detail), repeat station (RS) networks were founded in a lot of countries on the Earth. Regarding to the main task of RS measurements, almost the same requirements must be fulfilled during the fieldwork as in the GOs. Consequently, the environmental and instrumental expectations are pre-defined as well as the procedure of measurement which are generally absolute readings. After the reduction of the absolute measurements to the simultaneous record of the nearest GO is performed, the data can be represented to a relevant yearly mean value.

The present article shortly summarises the environmental and the instrumental background of geomagnetic absolute measurements on reoccupations of two different RS in Croatia. In 2010 tree-days-long reoccupations were performed on the selected stations with applying an on-site magnetic variometer.

One of the results of data processing pointed out the appearance of a geomagnetic sea-side effect on both of RSs. The phenomenon is the geomagnetic influence of the induced current system in the seawater near to the shore. The anomalous lateral currents are the consequence of the high conductivity contrast between the mainland and the (moving) salinity water and the induction effect of the fluctuating external geomagnetic field.

The calculation steps of the geomagnetic elements will be also detailed regarding the recorded samples of a presented dataset as well as the diagnostic values (i.e., offset value and misalignment error of fluxgate probe).

Specifications Table

**Table:** 

Subject	Geophysics
Specific subject area	Geomagnetism. Repeat station survey.
Data format	Raw, Analysed
Type of data	Table, Text files
Data collection	The most important files are the records of DIM (Declination and Inclination Magnetometer) and Overhauser magnetometer during re-occupation of two geomagnetic repeat stations (RS) near the Adriatic Sea. Additional files are the records of Hungarian RS measurement campaign in the same year. The results of the additional measurements can be a reference for the main dataset because those RSs are far away from the shore of the sea. Moreover, the spatial coverage of the dataset is especially high. During RS measurements, absolute measurements were performed. The DIM itself is suitable for measuring only the direction, i.e. the declination (*D*) and the inclination (*I*) of the geomagnetic field, but not its magnitude. The properly used DIM gives the mean absolute value of *D* and *I* for the observing period. Combined with a nuclear (i.e. Overhauser) magnetometer, DIM can describe all magnetic elements.
Data source location	In the years 2008, 2009, and 2010 the whole Croatian territory was covered by a dense Croatian Geomagnetic Network for Field Mapping (CGNFM) bringing better insights into the features of the magnetic field in Croatia. In the year 2010, the RS survey at Krbavsko polje (KRBP) and Sinjsko polje (SINP) was also performed. The Croatian dataset consists of all complete sets of absolute measurements of the two stations. In detail, latitude, longitude, and elevation of KRBP are 44.7° N, 15.6° E, 648 m, for SINP geographical coordinates are 43.6° N, 16.7° E, 296 m. In the year 2010 the Hungarian RS network was also surveyed. The name of the RS's, latitude and longitude are given below: Aggtelek 48.456 20.489 Bajásenye 46.799 16.409 Csátalja 46.039 18.972 Fertőd 47.666 16.895 Furta 47.101 21.462 Karos 48.338 21.742 Mezőcsát 47.827 20.835 Nagyszekeres 47.964 22.617
	Nyirád 47.018 17.461 Szank 46.545 19.699 Tápiógyörgye 47.316 19.971
Data accessibility	Repository name: DIM_sets Data identification number: DOI: 10.17632/hzphnd3p42.2 Direct URL to data: https://data.mendeley.com/datasets/hzphnd3p42/2

## 1 Value of the Data

*Why are these data valuable?*

• The result of the RS measurements is generally used for SV (Secular Variation) studies. On the other hand, the value of calculated geomagnetic elements is usable to update the latest geomagnetic field survey as well as for developing geomagnetic field models.

*How can these data be reused by other researchers?*

• The dataset is a real samples of a well documented RS measurements. Several scientific results were published because of the successful measurement campaign. This dataset improves the transparency of data processing. In this way all calculation steps of RS data processing can be test on the observed samples. On the other hand, the measurement in Adriatic region is influenced by effect of geomagnetic induction. The absolute measurements in Hungary are free from sea-side effect. In this way the dataset can be a reference for other observers.
• Generally, only the results of the absolute measurements are published. This dataset is one of the firsts where all readings are published by the observers.

## 2 Background

Variations of the Earth's magnetic field appear on a wide time scale. In terms of their origin, we distinguish between internal (induced by processes connected to the outer core of our planet and related to crustal anomalies) and external magnetic fields. In general, it can be said that changes of internal origin can be observed among phenomena lasting longer than a few months. Secular variation (SV) means changes with a period longer than one year. Geomagnetic observatories (GO) provide high-precision and continuous data for the study of temporal variations. To monitor the spatial changes, the so-called repeat station (RS) networks are used, since the density of observatories is sparse for the territorial assessment of SVs. Therefore, RS networks were established in different countries with the aim of periodically monitoring the secular magnetic variation.

During RS measurements, absolute measurements are performed. The RS data is taken at different times. To be the result comparative, the measurements must be subject to a temporal reduction of the continuous time series and the relevant yearly mean of the nearest GO. Different solutions were developed to use the proper geomagnetic conditions for the computation of the spatial difference [1].

## 3 Data Description

### 3.1 The description of the ASCII data files

*The name of the file:* AB (absolute measurement), date (yy,mm,dd)

*The extension:***sim** belongs to Sinsko Polje (SINP) station morning sets, **sie** the same station evening sets. **krm** belongs to Krbavsko Polje (KRBP) station morning sets, **kre** is the same station evening sets. The extensions in HUN_RS directory have no additional meanings.

**Table:** 

*Header:* Name_of_the_station; extension; Instrument; Date; Name_of_the_Observer1; Name_of_the_Observer2
*Data:*
The direction of azimuth mark (xxx° yy.yy') delta F (nT)
MIRA1 (xxx° yy.yy')
MIRA2 (xxx° yy.yy')
East Up (xxx° yy.yy') Time (UTC) (hh mm)
West Up (xxx° yy.yy') Time (UTC) (hh mm)
East Down (xxx° yy.yy') Time (UTC) (hh mm)
West Down (xxx° yy.yy') Time (UTC) (hh mm)
Nord Up (xxx° yy.yy') Time (UTC) (hh mm) Total field (nT)
South Down (xxx° yy.yy') Time (UTC) (hh mm) Total field (nT)
Nord Down (xxx° yy.yy') Time (UTC) (hh mm) Total field (nT)
South Up (xxx° yy.yy') Time (UTC) (hh mm) Total field (nT)
MIRA1 (xxx° yy.yy')
MIRA2 (xxx° yy.yy') etc...

*Note:* The names of samples are clearly defined in the following section.

## 4 Experimental Design, Materials and Methods

### 4.1 Croatian geomagnetic repeat station network (CGRSN)

After the establishment of the independent Republic of Croatia in the early 1990s, the geomagnetic field on the Croatian territory has been surveyed and monitored on the Croatian Geomagnetic Repeat Station Network, field mapping has been carried out through the Croatian Geomagnetic Network for Field Mapping (CGNFM), and finally in 2012 was established the geomagnetic observatory in Lonjsko polje (LON) [2].

The repeat station network was designed according to the IAGA standards and Magnetic Network in Europe (MagNetE) criteria considering spatial planning maps, historical data related to *F* anomalies of 1927.5, lithospheric models, and geological maps [3]. The repeat station network was primarily designed to cover the national territory evenly with 8 stations. Whenever possible, the RSs were planned at macrolocations distant at least 10 km from the sea in order to fulfill the homogeneous electric conductivity criterion [4].

The network was established and surveyed in the summer of 2004. Specific chosen microlocations were previously checked for the absence of civilian noise by observing and analysing the records of the scalar total field *F*. Four different methods of gradient determination using only one PPM (Proton Precession Magnetometer) gradiometer were developed and used during RS establishment, namely: vertical gradient above the RS, quick area 10 m around the RS, inner-grid method around the RS (2 m x 2 m) and outer-grid method around the RS (10 m x 10 m). The maximum gradient value was set to 3 nT/m and only when all gradiometry methods have reached acceptable results, the location was accepted as an RS. Details about Croatian RS network establishment may be found in [3] and [5].

According to the recommendations [6] and later confirmed criteria by [7], around each RS at distances whenever possible greater than 200 m, were established several orientation points (azimuth marks). Coordinates of the RS and reference mark were determined by GPS (Global Positioning System) static relative positioning providing cm-level (or even better) accuracy level. During the summer of 2008, CGRSN was enlarged by two additional stations on the Adriatic islands bringing the network to its current shape given in [8] or [9].

In the years 2008, 2009, and 2010 the whole Croatian territory was covered by a dense Croatian Geomagnetic Network for Field Mapping (CGNFM) bringing better insights into the features of the magnetic field in Croatia. The subsequent analysis of the magnetic field has proven that the RSs were established not far away from their optimum positions [8].

Owing to a fruitful cooperation between the University of Zagreb – Faculty of Geodesy and the Geomagnetic observatory Tihany in Hungary, in 2010 was launched the ‘*Joint Croatian-Hungarian Geomagnetic Repeat Stations Survey and Joint Geomagnetic Field Model*’ project. One of the project's outcomes was the joint geomagnetic repeat stations survey carried out in the summer of 2010 on three RS in Croatia: KRBP, SINP, and PALA [9]*.* During each repeat station occupation lasting for three and half days, several morning and evening absolute observation set sessions were performed enabling determination and monitoring of dIdD (delta Declination/ delta Inclination) baseline values.

The joint survey in 2010 was carried out with two field teams, consequently, almost simultaneous surveys took place on the pairs of repeat stations: KRBP and POKU, SINP and KONA, enabling the reduction of surveyed geomagnetic elements to the neighbouring repeat station running an onsite variometer [10]. During the survey in the summer of 2010, static GNSS (Global Navigation Satellite System) observations were carried out on all RS stations and associated azimuth marks, enabling thorough analysis and determination of azimuths in a homogeneous geodetic reference frame [7,10]. Azimuth determination by methods of geodetic astronomy and subsequent comparison with azimuth value determined by GNSS method on POKU RS has shown a great level of matching and confirmed the reliability of both azimuth determination methods [7].

### 4.2 The description of the elements of the geomagnetic field

**Fig. 1:**
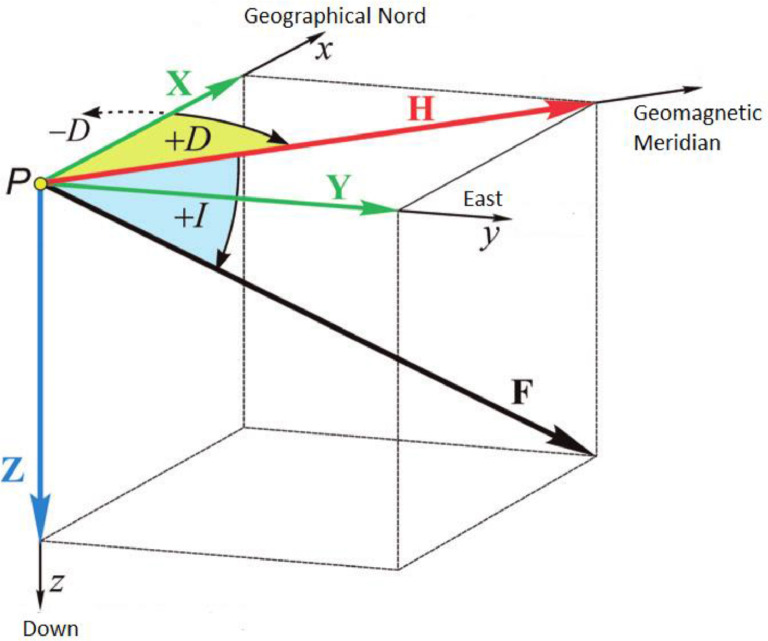
The geomagnetic components. (After [11].).

The magnetic field of the Earth is changing in time and space. The geomagnetic field is a vector field that can be given by three elements depending on the reference system. The quantities used to describe the magnetic field are defined with respect to the geographic coordinate system (Fig. 1). The declination (*D*) is the angle between the true North and geomagnetic North, positive to the East. The inclination (*I*) is the angle which is made by the magnetic vector to the horizontal plane. The total field (*F*) is the intensity of the field vector (scalar value). Other widely used magnetic components are horizontal intensity (*H* – the projection of the field vector to the horizontal plane), vertical intensity (*Z* – the vertical component of the field), north component (*X* – component pointing to the true North in the horizontal plane), East component (*Y* – component pointing to the East in the horizontal plane).

The international standard for global distribution of normal values of the magnetic field is published by the IAGA every five years. It is called the International Geomagnetic Reference Field (IGRF).

Our dataset consists of complete set of geomagnetic absolute measurements so our description will focus on this measurement technique.

## 5 Geomagnetic Absolute Measurements

We can accept a geomagnetic record as a magnetic absolute measurement, in the course of which the measurement of the magnetic field is traced back to the measurement of other basic physical quantities (i.e., time, angle). In addition, we can calculate (consequently also eliminate) some errors that may affect the measurement result [12,13].

Presently there are two types of magnetometers that can fulfill the requirements of an absolute measurement: the nuclear magnetometers and the Declination and Inclination Magnetometer (DIM). There are developments also to perform automatic absolute measurements (i.e. AutoDIF), but they have not been used for fieldwork up till now.

### 5.1 Nuclear magnetometers

The different nuclear magnetometers (proton precession, Overhauser, potassium, cesium etc.) measure the magnitude of ambient geomagnetic field based on quantum mechanical evidence.

In the case of standard Proton or Overhauser magnetometers the protons (in the case Overhauser effect the unbound electrons also) are polarized to a higher thermal equilibrium (which is practically an excited spin level of the particles) in a hydrogen-rich fluid. After an impulsive EM signal, they return to a steady state, and during the process the protons perform a decaying gyromagnetic precession on a discrete frequency. The frequency of the radiant electromagnetic wave is proportional to the ambient magnetic field. The results of a magnetic measurement do not depend on any other physical parameters, only on the value of the gyromagnetic constant of protons, which is a universal constant (*g_p_*=2.675153362×10^8^ *T* ^−^^1^s^−1^). During the measurement, the angular velocity of the gyromagnetic precession must be determined, so we can trace back the magnetic observations to the measurement of time.

(1) $$\omega =Bg_{p},$$

where *B* indicates the total intensity of the magnetic field, *ω* is the measured angular velocity of the precession of protons, and *g_p_* is the value of the gyromagnetic constant.

In normal operation of the Proton or Overhauser magnetometers, the record is free from calibration errors and the offset of the device is always zero (in the case of different total field instruments [i.e. Cesium magnetometers] the output value can be the function of the orientation of the probe). We used Overhauser magnetometers during the RS campaign.

### 5.2 The DIM device

The DIM itself is suitable for measuring only the direction, i.e. the declination (*D*) and the inclination (*I*) of the geomagnetic field, but not its magnitude. The properly used DIM gives the mean absolute value of *D* and *I* for the observing period. Combined with a nuclear magnetometer DIM can describe all magnetic elements.

The DIM consists of a nonmagnetic theodolite and a one-axis fluxgate magnetometer. The sensor of the fluxgate magnetometer is mounted on the telescope of the theodolite.

The following expectations are required for the theodolite:

- The vertical scale readings of the device must be mechanically gravity-controlled. (Built-in compensator is expected.)
- Magnetic hygiene of the instrument (everywhere less than 1 nT at 1 cm distance from the surface of the instrument) makes it possible to carry out valid measurements.
- The resolution of the angle reading is 0.1′ or better.
- All mechanical and optical functions of the theodolite must be well adjusted.
- The one-component magnetometer must fulfill:
- The magnetometer yields 1 nT resolution field reading or better.
- The offset of the device must be stable during a complete set of absolute measurements.

Moreover, the small magnitude of the offset (less than 10 nT) helps the observer to perform the measurement. Further advance can be achieved when the magnetic axis of the sensor fixed on the theodolite is carefully adjusted to the optical axis of the telescope within some tens of arc seconds.

## 6 Preparation for an Absolute Measurement on RS

During RS reoccupation the purpose of absolute measurements is to determine the magnetic elements of the site. The measurements are taken on a pier (if it exists) or on a non-magnetic tripod. The measured point of RS and its surrounding, as the instrument itself, are supposed to be free of any magnetic contamination. Care must be taken to avoid any influence from magnetic objects (cars, equipment etc.) during the measurement procedure. The observer himself/herself also must be clean magnetically, magnetic glasses, pens, and watches must be removed from the vicinity of the DIM. The safety distance for every object should be determined by the measurement of its magnetic influence.

The accuracy of the absolute measurement depends even on the disturbance level (the time derivative) of the field. Measurements, that are taken during highly disturbed, stormy periods can lead to unacceptable results.

For the determination of the declination in addition to measurements made by the DIM, a reference direction is required. The easiest way is to use a reference mark (also called azimuth mark). It is necessary to know the azimuth of this mark (viewing from the absolute pier) with an accuracy of 0.1 arc minute in the field. The horizontal error of the positioning of the theodolite above the pier mark and the distance of the reference mark influence the accuracy of the declination.

## 7 Absolute Measurement Process

### 7.1 The null reading method

In the following section, the procedure of an absolute measurement according to the null method will be given.

As the mechanical axes of the theodolite, the optical axis of the telescope and the magnetic axis of the fluxgate sensor always have some misalignment error, moreover, the theodolite may have further mechanical errors (collimation errors, non-orthogonality errors of the horizontal and vertical axis, etc.), and as the offset of the electronics is not zero, the declination/inclination of the field cannot be determined from a single reading of the horizontal/vertical circle. However, most of these errors are eliminated by the measuring process, using four position readings for each element [12].

After the levelling of the theodolite on the pier or on the tripod, the adjustment of the horizontal circle of the instrument is advised. Following a comfortable procedure, we use a −90° difference for the sensor up position. In details: With the telescope pointing to the azimuth mark, adjust the horizontal circle approximately to Az-90°. If Az-90°<0°, add 360°.

#### 7.1.1 Observation of declination

##### 7.1.1.1 Determination of the position of the horizontal circle with azimuth mark

Point the telescope to the azimuth mark (sensor up) and the observer reads the horizontal circle. The registered value is *MIRA1.* Invert the telescope to have the sensor on the bottom (sensor down position) and the observer reads the horizontal circle again. The registered value is *MIRA2.*

##### 7.1.1.2 East up position

In this position the sensor is on the telescope and the vertical index is exactly 90°. The telescope points roughly to the East. After fine adjustment of the alidade, the observer slowly searches for the null position (null reading on the display of the electronics). At this moment the observer registers the time (*t_Eup_*) always in UTC (Universal Time Coordinated) and the actual value of the horizontal circle (*A_1_* – East up).

##### 7.1.1.3 West up position

**Fig. 2:**
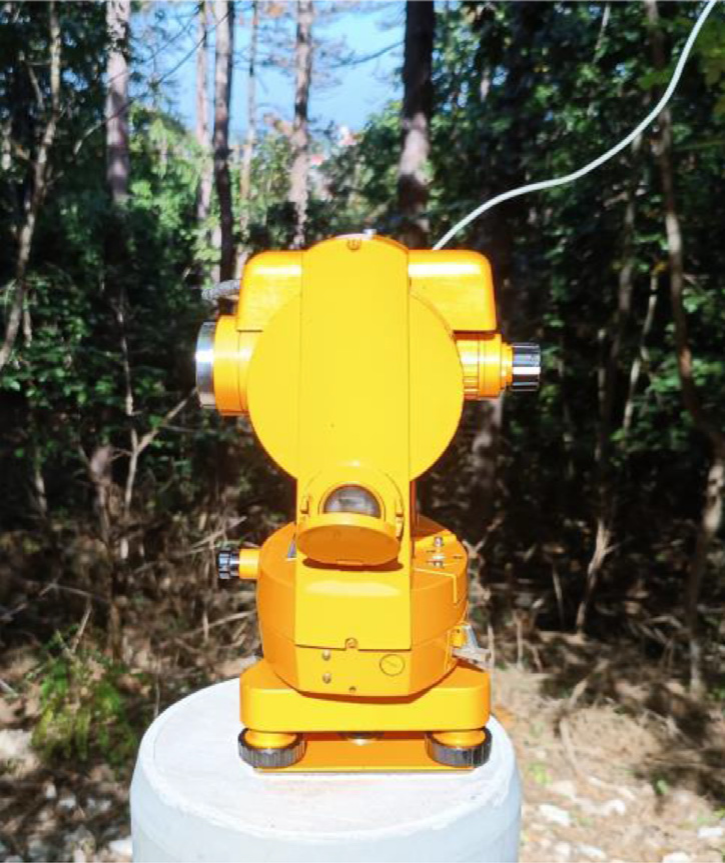
West up – (A_2_) position of DIM device on the outer pillar of Tihany Geomagnetic Observatory. The theodolite is a steal-free Zeiss 020 A equipped with DMI D&I fluxgate probe.

After East up position by rotation of the alidade with approximately 180° we get the telescope points roughly to the West (see Fig. 2 for the details). The vertical index must be exactly 90° again. The observer adjusts the alidade slowly searching for the null position. The observer registers the time at the moment of null reading (*t_Wup_*) and the recorded value of the horizontal circle (*A_2_* – West up).

##### 7.1.1.4 East down position

From the previous (*A_2_* – West up) position we can reach the following by turning the telescope to have the sensor down. The vertical index must be exactly 270° and the telescope points roughly to the East. After fine adjustment, the observer searches for the null position again. The time of the null reading is (*t_Edn_*) and the recorded value of the horizontal circle is (*A_3_* – East down).

##### 7.1.1.5 West down position

The direction of the telescope is more or less opposite to the previous (*A_3_* – East down) reading. The vertical index must be exactly 270° again. The observer adjusts the alidade slowly searching for the null position. The observer registers the time at the moment of null reading (*t_Wdn_*) and the recorded value of the horizontal circle (*A_4_* – West down).

##### 7.1.1.6 Calculation of the declination

The *Az* value is the azimuth of the reference mark.

*M* is the average of the measurements of reference mark sighting corrected with 180°:

(2) $$M=\frac{\text{MIRA}1+\text{MIRA}2}{2}-180^{\circ },$$

We can calculate the angle of the magnetic meridian (*A*) in the theodolite reference frame from the four declination readings:

(3) $$A=\frac{A_{1}+A_{2}+A_{3}+A_{4}}{4}+m\cdot 90^{\circ },$$

where *m*=-/+1 for East/West declination.

The mean declination (regarding *t_Eup_, t_Edn_, t_Wup_* and *t_Wdn_* moments) is given by:

(4) $$D_{abs}=A+A_{Z}-M.$$

#### 7.1.2 Observation of the inclination

##### 7.1.2.1 North up position

**Fig. 3:**
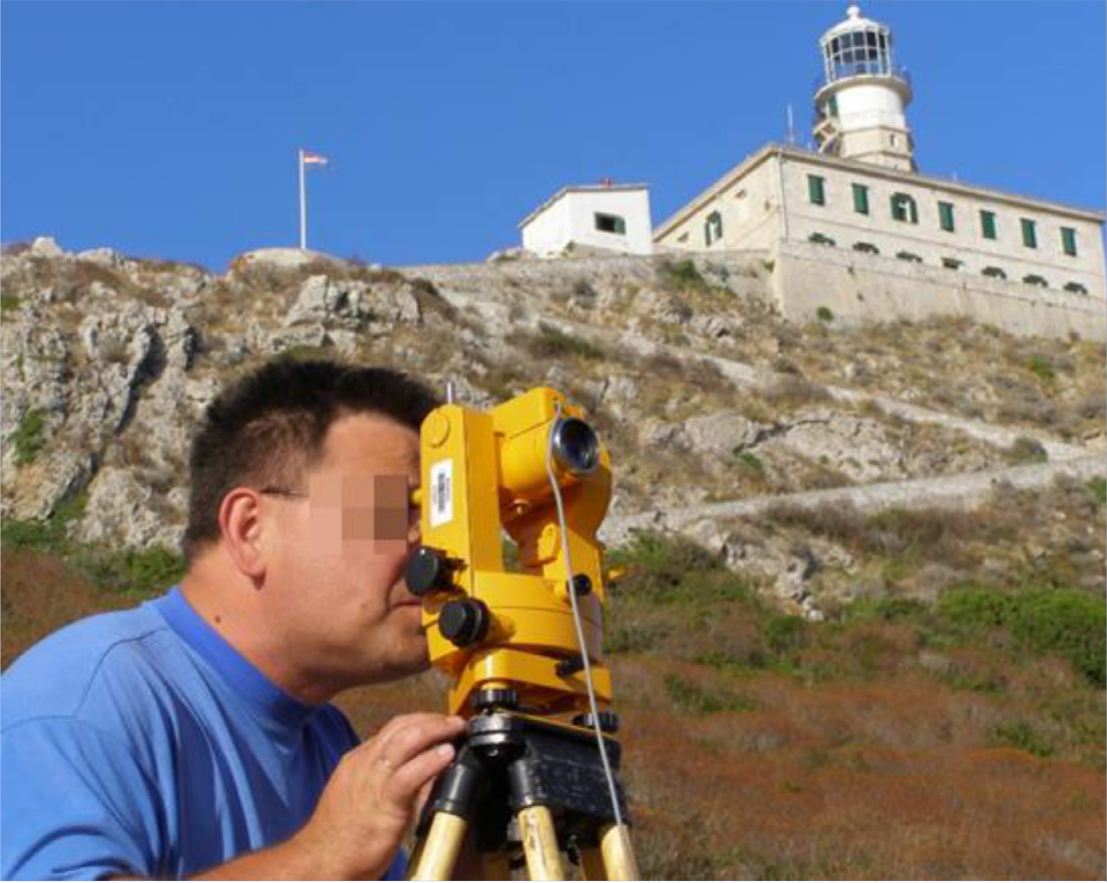
Observation of the Nord up – (V_1_) position during the reoccupation of Palagruža (PALA) RS (a morning set). The DIM instrument is identical to one which is presented in Fig. 2. Applying the tripod, the DIM was installed over the marked RS point.

In this position, the telescope has the sensor up again (see Fig. 3 for the details). The observer adjusts the telescope in the geomagnetic meridional plane, so the horizontal circle setting is exactly *A* + 270° The telescope should be nearly orthogonal to the total field vector again. With fine adjustment, the observer searches for the null position. The time at the moment of null reading is (*t_Nup_*) and the momentary total field value is (*F_Nup_*). The registered value of the vertical circle is (*V_1_* – North up).

##### 7.1.2.2 South down position

After the North up position with the rotation of the telescope by approximately 180°, we get the telescope pointing roughly to the South. With fine adjustment, the observer searches for the null position. The registered time at the moment of null reading is (*t_Sdn_*). The recorded momentary total field value (*F_Sdn_*). The registered value of the vertical circle is (*V_2_* – South down).

##### 7.1.2.3 North down position

The observer rotates the alidade at exactly 180° (*A* + 90°, telescope in the meridional plane). The telescope should be nearly orthogonal to the total field vector again. With fine adjustment, the observer searches for the null position. The time at the moment of null reading is (*t_Ndn_*) and the momentary total field value is (*F_Ndn_*). The registered value of the vertical circle is (*V_3_* – North down).

##### 7.1.2.4 South up position

After turning the telescope, it points roughly to the South. With fine adjustment, the observer searches for the null position. The registered time at the moment of null reading is (*t_Sup_*) and the momentary total field value is (*F_Sup_*). The registered value of the vertical circle is (*V_4_* – South up).

The mean inclination is the average of the four vertical circle readings taken during the inclination observation process, corrected:

(5) $$I_{abs}=\frac{V_{1}+V_{2}+V_{3}+V_{4}}{4}+90^{\circ }.$$

If more sets are taken, the whole procedure must be repeated from the beginning.

Note: There are different measurement methods to perform an absolute measurement with DIM. The residual method [12] is also used worldwide. This method uses the non-zero output of D&I fluxgate also in the protocol.

Meantime a new procedure was introduced based on arbitrarily distributed DI-fluxgate theodolite orientations [14].

In the recently published dataset only the null method was used.

## 8 Calculation of the Geomagnetic Vector Components and the Estimation of Misalignment Errors and Offset

Generally, the goal of a DIM measurement is to obtain all the elements of the geomagnetic field, and therefore the DIM is combined with a scalar magnetometer. The simultaneous use of two instruments gives rise to an additional problem as all the measurements must be referred to the same point (the place of the theodolite). That means that the total field difference between the two places *dF* (the place of the scalar device F_REC_ and the theodolite F_DIM_) has to be measured.

(6) $$dF=F_{DIM}-F_{REC}$$

The mean total field is calculated as the average of the four readings corrected with site difference:

(7) $$A=\frac{F_{Nup}+F_{Sdn}+F_{Ndn}+F_{Sup}}{4}+dF.$$

Horizontal (*H*), vertical (*Z*), North (*X*), and East (*Y*) intensities can be expressed as:

(8) $$H_{abs}=F\cdot \text{cos}\left(I_{abs}\right),$$

(9) $$Z_{abs}=F\cdot sin\left(I_{abs}\right),$$

(10) $$X_{abs}=H_{abs}\cdot \text{cos}\left(D_{abs}\right),$$

(11) $$Y_{abs}=H_{abs}\cdot sin\left(D_{abs}\right).$$

The measured absolute values are free from every possible instrumental error if all predefined expectations are fulfilled. Namely, the following parameters are expected to be zero:

- offset value,
- scale factors,
- orientation errors (both in vertical and horizontal directions),
- orthogonality errors of the measured components.

Nowadays there is no other way to validate the reliability of the DIM instruments, only the comparison of different devices.

The measurement of the DIM could be influenced by different errors. These errors are eliminated by the measurement method which is presented in Section 4 [12]. Moreover, we can determine the magnitude of the eliminated errors.

The fluxgate sensor mounted on the telescope has two misalignment errors, one in the horizontal (*δ*) and another one in the vertical plane (*ε*). The reference direction is the optical axis of the theodolite. The horizontal misalignment can be estimated from the four horizontal readings taken during declination observation:

(12) $$\delta =\frac{A_{1}+A_{2}-A_{3}-A_{4}}{4}.$$

The vertical misalignment can be estimated from the four vertical readings taken during inclination observation:

(13) $$\varepsilon =-\frac{V_{1}+V_{2}+V_{3}+V_{4}}{4}+180^{\circ },$$

The offset *(S)* of the fluxgate device estimated from both sequences:

(14) $$S_{0D}=\left(A_{1}-A_{2}+A_{3}-A_{4}\right)\cdot \frac{H_{\text{abs}}}{4}\cdot \frac{180}{\pi },$$

(15) $$S_{0}=-\left[\left(V_{1}-V_{2}-V_{3}+V_{4}\right)/4+90\right]\cdot F\cdot 180/\pi$$

where S_0D_ is the offset measured during the declination sequence and *S_0_* is the calculated offset regarding the inclination readings. This scalar value is the output of the D&I fluxgate device in a zero magnetic field.

## 9 The Results of the Calculation Processes

During the RS campaign, we used Zeiss 020A steal-free theodolite which was equipped with DMI D&I fluxgate probe. The Zeiss 020A device makes it possible to estimate the circle readings of the theodolite to 0.1′. The resolution of the one-component fluxgate magnetometer is 0.1 nT.

We performed the calculation steps presented in Eqs. (2)-(15). The results of these calculations are presented in Table 1 for KRBP RS and in Table 2 for SINP RS.

**Table 1:** The results of geomagnetic absolute measurements on KRBP RS. The “DoY” represents the daynumber of the year 2010. The “hh” marks the hour of the measurement in UTC. The “mm” is the minute of South down readings. The calculated parameters are defined in (4), (5) and (8)-(15) equations, respectively.

Time (2010)			D_abs_		I_abs_		Calculated geomagnetic components				Misalig. Err.		Offsets	
DoY	hh	mm	(°)	(‘)	(°)	(‘)	X_abs_ (nT)	Y_abs_ (nT)	Z_abs_ (nT)	H_abs_ (nT)	δ(‘)	ε(‘)	S_0D_ (nT)	S_0_ (nT)
201	5	14	2	53.62	61	13.75	22,696.8	1147.3	41,387.8	22,725.7	0.90	−0.55	4.0	4.8
201	5	32	2	53.67	61	13.67	22,697.0	1147.6	41,386.2	22,726.0	0.90	−0.67	5.0	3.8
201	5	42	2	53.72	61	13.85	22,694.6	1147.8	41,386.8	22,723.6	0.80	−0.60	4.0	3.4
201	6	2	2	54.17	61	14.15	22,690.3	1150.6	41,387.8	22,719.4	0.80	−0.45	4.6	4.8
201	6	20	2	54.12	61	14.45	22,685.9	1150.0	41,388.2	22,715.0	1.00	−0.50	3.3	4.1
201	6	38	2	54.00	61	14.83	22,680.1	1148.9	41,388.4	22,709.2	1.03	−0.57	4.5	4.5
201	16	20	2	49.57	61	14.60	22,686.8	1120.0	41,391.4	22,714.4	0.90	−0.70	1.3	3.4
201	16	39	2	49.72	61	14.50	22,687.8	1121.0	41,390.6	22,715.5	0.75	−0.65	3.0	2.1
201	17	0	2	49.40	61	14.43	22,688.7	1118.9	41,389.9	22,716.3	1.03	−0.62	−0.2	3.1
201	17	13	2	49.77	61	14.58	22,685.8	1121.2	41,389.1	22,713.5	0.80	−0.72	2.0	3.1
201	17	30	2	49.82	61	14.43	22,687.4	1121.6	41,387.8	22,715.1	0.70	−0.63	2.3	2.4
201	17	45	2	49.92	61	14.40	22,687.4	1122.3	41,387.1	22,715.1	0.75	−0.60	3.0	2.7
202	8	2	2	54.70	61	14.63	22,677.1	1153.4	41,377.7	22,706.4	0.58	−0.83	2.8	3.1
202	8	15	2	54.40	61	14.33	22,680.7	1151.6	41,375.5	22,709.9	0.53	−0.88	1.8	1.7
202	8	33	2	53.85	61	14.17	22,683.4	1148.1	41,375.8	22,712.5	0.53	−0.98	0.5	1.7
202	8	48	2	53.45	61	14.05	22,685.5	1145.5	41,375.8	22,714.4	0.63	−1.00	1.2	0.7
202	17	13	2	50.35	61	14.60	22,682.0	1124.9	41,383.3	22,709.9	0.48	−0.55	0.8	1.4
202	17	26	2	50.72	61	14.53	22,683.4	1127.4	41,383.9	22,711.4	0.40	−0.68	0.3	1.7
202	17	48	2	51.00	61	14.43	22,686.0	1129.3	41,386.0	22,714.1	0.33	−0.57	1.2	1.0
202	18	0	2	50.87	61	14.43	22,686.3	1128.5	41,386.3	22,714.3	0.40	−0.58	1.3	0.3
203	7	15	2	53.85	61	16.70	22,655.6	1146.7	41,397.2	22,684.6	0.58	−1.70	3.1	2.7
203	7	27	2	53.92	61	15.78	22,665.6	1147.7	41,389.1	22,694.7	0.50	−0.82	2.6	3.1
203	7	45	2	53.72	61	15.95	22,662.1	1146.2	41,387.5	22,691.1	0.55	−0.70	2.6	2.7
203	7	58	2	54.52	61	15.97	22,660.7	1151.4	41,386.2	22,690.0	0.40	−0.82	3.0	1.0
203	8	21	2	52.45	61	16.00	22,659.4	1137.6	41,383.3	22,688.0	0.28	−0.80	2.1	0.7
203	14	35	2	49.12	61	14.55	22,686.2	1117.0	41,388.7	22,713.7	0.05	−0.50	2.3	2.1
203	14	47	2	49.25	61	14.63	22,685.1	1117.7	41,388.9	22,712.6	0.08	−0.52	1.2	1.7
203	16	42	2	51.70	61	14.95	22,680.4	1133.7	41,391.1	22,708.7	0.28	−0.55	0.8	2.7
203	16	55	2	51.52	61	15.25	22,675.7	1132.3	41,391.0	22,703.9	0.25	−0.55	0.3	2.7
203	17	10	2	51.62	61	15.17	22,676.5	1133.0	41,390.4	22,704.8	0.30	−0.53	1.7	1.7
203	17	25	2	51.67	61	15.00	22,678.7	1133.5	41,389.5	22,707.1	0.25	−0.40	2.3	2.1
203	17	38	2	51.47	61	14.98	22,679.1	1132.1	41,389.4	22,707.4	0.35	−0.32	2.0	2.4
203	18	10	2	50.85	61	14.83	22,680.8	1128.1	41,387.7	22,708.8	0.43	−0.23	2.8	2.4
204	5	41	2	54.95	61	14.15	22,692.5	1155.8	41,392.3	22,721.9	0.48	−0.10	2.5	2.7
204	5	52	2	54.82	61	14.18	22,692.4	1155.0	41,392.8	22,721.8	0.45	−0.12	2.3	3.1
204	6	3	2	54.85	61	14.28	22,691.5	1155.1	41,394.1	22,720.9	0.48	−0.08	2.5	3.8
204	6	15	2	55.00	61	13.98	22,694.8	1156.3	41,391.5	22,724.2	0.48	−0.52	2.5	3.8
204	6	31	2	55.32	61	14.18	22,691.7	1158.3	41,391.8	22,721.2	0.55	−0.47	2.0	3.8

**Table 2:** The results of geomagnetic absolute measurements on SINP RS. The “DoY” represents the daynumber of the year 2010. The “hh” marks the hour of the measurement in UTC. The “mm” is the minute of South down readings. The calculated parameters are defined in (4), (5) and (8)-(15) equations, respectively.

Time (2010)			D_abs_		I_abs_		Calculated geomagnetic components				Misalig. Err.		Offsets	
DoY	hh	mm	(°)	(‘)	(°)	(‘)	X_abs_ (nT)	Y_abs_ (nT)	Z_abs_ (nT)	H_abs_ (nT)	δ(‘)	ε(‘)	S_0D_ (nT)	S_0_ (nT)
204	18	7	2	51.62	60	28.58	23,182.6	1158.3	40,986.6	23,211.5	0.48	−0.58	1.2	1.7
204	18	18	2	51.15	60	28.62	23,182.6	1155.1	40,987.7	23,211.4	0.35	−0.52	2.0	1.7
204	18	29	2	51.32	60	28.58	23,183.7	1156.4	40,988.3	23,212.5	0.38	−0.58	0.8	1.0
205	6	1	2	56.12	60	29.08	23,174.1	1188.3	40,988.2	23,204.6	0.38	−0.57	2.5	3.8
205	6	11	2	56.32	60	29.08	23,174.6	1189.7	40,989.1	23,205.1	0.53	−0.63	1.5	3.1
205	6	22	2	56.35	60	29.13	23,173.8	1189.8	40,989.1	23,204.3	0.40	−0.63	1.3	3.1
205	6	35	2	56.37	60	29.23	23,172.4	1189.9	40,989.5	23,202.9	0.28	−0.52	2.2	1.7
205	6	48	2	56.32	60	29.28	23,171.6	1189.5	40,989.5	23,202.2	0.28	−0.52	1.5	1.7
205	7	3	2	56.12	60	29.20	23,172.6	1188.2	40,989.0	23,203.1	0.18	−0.45	2.2	2.1
205	17	17	2	53.22	60	28.88	23,176.3	1168.8	40,984.8	23,205.8	0.13	−0.27	1.5	3.1
205	17	31	2	52.80	60	28.98	23,175.3	1165.9	40,985.5	23,204.6	0.30	−0.18	3.7	2.4
205	17	49	2	52.47	60	29.03	23,175.0	1163.7	40,986.1	23,204.2	0.43	−0.23	3.5	3.1
205	18	4	2	52.70	60	29.25	23,172.0	1165.1	40,987.2	23,201.2	0.55	−0.35	4.0	3.4
205	18	23	2	52.30	60	29.13	23,174.3	1162.5	40,987.6	23,203.5	0.55	−0.23	3.4	3.1
206	6	9	2	56.20	60	29.78	23,164.9	1188.4	40,991.4	23,195.4	0.60	−0.33	4.7	3.8
206	6	24	2	56.17	60	29.98	23,161.8	1188.0	40,991.4	23,192.2	0.63	−0.43	3.5	4.4
206	6	38	2	56.30	60	30.15	23,159.4	1188.7	40,992.1	23,189.8	0.65	−0.30	4.0	4.2
206	6	52	2	56.32	60	30.30	23,157.0	1188.8	40,992.2	23,187.5	0.68	−0.31	4.2	4.0
206	7	10	2	55.80	60	30.38	23,156.0	1185.2	40,992.1	23,186.3	0.55	−0.37	3.7	1.7
206	17	27	2	53.25	60	29.68	23,168.8	1168.6	40,993.8	23,198.3	0.60	−0.23	5.4	5.8
206	17	47	2	53.25	60	29.40	23,172.8	1168.8	40,993.2	23,202.3	1.10	−0.20	2.4	4.8
206	18	2	2	52.85	60	29.35	23,173.2	1166.1	40,992.2	23,202.5	0.75	−0.15	2.7	5.5
206	18	14	2	53.15	60	29.45	23,171.0	1168.1	40,991.3	23,200.4	0.75	−0.15	3.0	5.5
207	6	16	2	56.05	60	29.98	23,162.4	1187.2	40,992.5	23,192.9	0.85	−0.43	5.1	5.1
207	6	29	2	56.22	60	30.02	23,161.4	1188.3	40,992.1	23,191.8	0.68	−0.37	5.6	5.1
207	6	44	2	55.45	60	30.05	23,161.0	1183.1	40,991.7	23,191.2	1.05	−0.45	5.7	6.2
207	6	59	2	55.65	60	30.20	23,158.0	1184.3	40,990.6	23,188.2	0.75	−0.35	4.7	4.1
207	7	18	2	56.25	60	30.30	23,154.8	1188.2	40,988.2	23,185.3	0.85	−0.40	3.0	4.1
207	17	9	2	52.57	60	29.58	23,169.2	1164.1	40,991.3	23,198.4	0.83	−0.23	4.9	5.1
207	17	21	2	52.70	60	29.58	23,169.0	1164.9	40,991.0	23,198.3	1.10	−0.22	5.1	5.1
207	17	32	2	52.57	60	29.50	23,170.1	1164.1	40,990.7	23,199.3	0.78	−0.30	4.2	4.8
207	17	48	2	52.22	60	29.53	23,169.2	1161.7	40,989.7	23,198.3	0.78	−0.17	4.6	5.1
207	18	7	2	52.05	60	29.50	23,169.3	1160.5	40,989.0	23,198.3	0.85	−0.30	3.4	4.8

We performed the same calculations for two Hungarian RSs order to present a real reference. The DIM instrument was the same during both reoccupations. The results of these calculations are presented in Table 3 for Szank RS and in Table 4 for Bajánsenye RS.

**Table 3:** The results of geomagnetic absolute measurements on Szank RS. The “DoY” represents the daynumber of the year 2010. The “hh” marks the hour of the measurement in UTC. The “mm” is the minute of South down readings. The calculated parameters are defined in (4), (5) and (8)-(15) equations, respectively.

Time (2010)			D_abs_		I_abs_		Calculated geomagnetic components				Misalig. Err.		Offsets	
DoY	hh	mm	(°)	(‘)	(°)	(‘)	X_abs_ (nT)	Y_abs_ (nT)	Z_abs_ (nT)	H_abs_ (nT)	δ(‘)	ε(‘)	S_0D_ (nT)	S_0_ (nT)
301	13	9	3	55.20	63	15.40	21,650.9	1483.6	43,067.8	21,701.7	1.00	−0.10	4.4	5.5
301	13	20	3	55.65	63	15.45	21,650.3	1486.4	43,068.6	21,701.3	0.80	−0.10	4.7	6.4
301	13	44	3	56.33	63	15.07	21,656.0	1491.1	43,068.8	21,707.3	0.68	−0.18	5.2	5.5
301	13	57	3	56.30	63	15.12	21,655.7	1490.9	43,069.7	21,706.9	0.55	−0.08	4.4	4.4
301	14	31	3	56.85	63	15.37	21,651.4	1494.1	43,069.4	21,702.9	0.15	0.07	6.0	6.7
301	14	41	3	56.78	63	15.22	21,653.4	1493.7	43,068.6	21,704.8	0.18	−0.03	6.8	6.8
301	15	11	3	57.90	63	15.15	21,653.6	1500.9	43,067.7	21,705.6	0.00	0.25	6.3	6.9
301	15	19	3	57.65	63	15.12	21,654.2	1499.3	43,067.8	21,706.0	0.15	0.12	5.4	5.3
302	6	28	3	59.48	63	14.88	21,659.0	1511.2	43,071.3	21,711.7	−0.13	0.28	7.7	6.7
302	6	39	3	59.73	63	14.88	21,658.8	1512.8	43,071.0	21,711.6	−0.38	0.33	6.8	6.7
302	6	50	4	0.00	63	14.98	21,657.3	1514.4	43,071.5	21,710.2	−0.60	0.47	8.8	8.1
302	7	5	4	0.43	63	14.90	21,658.2	1517.2	43,071.3	21,711.3	−0.73	0.40	8.4	7.8
302	7	15	4	0.28	63	14.95	21,657.4	1516.2	43,071.2	21,710.4	−0.73	0.40	9.0	9.8

**Table 4:** The results of geomagnetic absolute measurements on Bajánsanye RS. The “DoY” represents the daynumber of the year 2010. The “hh” marks the hour of the measurement in UTC. The “mm” is the minute of South down readings. The calculated parameters are defined in (4), (5) and (8)-(15) equations, respectively.

Time (2010)			D_abs_		I_abs_		Calculated geomagnetic components				Misalig. Err.		Offsets	
DoY	hh	mm	(°)	(‘)	(°)	(‘)	X_abs_ (nT)	Y_abs_ (nT)	Z_abs_ (nT)	H_abs_ (nT)	δ(‘)	ε(‘)	S_0D_ (nT)	S_0_ (nT)
341	14	8	3	8.50	63	17.15	21,566.9	1183.8	42,919.1	21,599.4	−0.75	−0.20	4.7	3.5
341	14	20	3	8.52	63	16.77	21,572.4	1184.2	42,918.5	21,604.9	−0.88	−0.17	4.6	6.6
341	14	32	3	8.42	63	16.60	21,575.2	1183.7	42,918.5	21,607.7	−0.88	−0.10	4.9	6.3
341	14	43	3	8.02	63	16.65	21,574.1	1181.2	42,917.6	21,606.4	−0.73	−0.10	4.6	6.3
341	14	58	3	8.45	63	16.72	21,572.6	1183.8	42,917.2	21,605.1	−0.55	−0.17	5.0	5.9
341	15	9	3	8.60	63	16.45	21,577.2	1185.0	42,917.9	21,609.7	−0.30	−0.10	4.1	5.6
342	7	43	3	11.00	63	16.02	21,581.1	1200.3	42,914.1	21,614.5	0.55	−0.02	6.6	4.5
342	7	54	3	10.60	63	16.02	21,580.8	1197.7	42,913.2	21,614.0	0.50	−0.07	3.5	3.1
342	8	6	3	10.50	63	16.20	21,577.9	1197.0	42,912.8	21,611.1	0.50	−0.05	4.7	4.2
342	8	16	3	10.57	63	16.42	21,574.6	1197.2	42,913.3	21,607.8	0.38	−0.03	5.5	5.9

## 10 The Importance and the Usage of the Geomagnetic Absolute Measurements

The absolute measurements are the only way to control the base values of variometers. The reliability of the geomagnetic recording system is determined by its long-term baseline stability. The geomagnetic observatories must perform absolute measurements at least with one-week frequency.

The activity of RS measurement is also based on the geomagnetic absolute measurement. There is no other relevant solution on how to determine geomagnetic elements during the reoccupation of an RS.

The presented dataset was used also for the baseline determination of the on-site variometer. Previous studies also reported that the measured baseline fluctuations during the RS survey have a natural reason [15].

The peculiarity of the survey near the Adriatic Sea was the fact that it was the first RS survey carried out using an onsite variometer, namely the dIdD. The first results of that survey were published in [16,10], and [9]. By analysis of the variometer baseline components, it was readily visible their temporal changes that were attributed to significant conductivity contrast between the Adriatic Sea and the mainland. That conductivity contrast can result in spatial field variation in small spatial and temporal scales, even in large distances. Those assumptions were later on elaborated in [17] and well as in [18,19] and [11].

## 11 Summary

In a recent paper we presented the most important circumstances of two RS measurements in the Adriatic region as well as the applied measurement methods in detail. Additionally, the results of two Hungarian RS absolute measurement are also referred. The calculated geomagnetic elements and parameters of the geomagnetic absolute measurements are also presented. The published dataset fulfills all requirements of present-day technical and scientific trends.

## Limitations

Not applicable.

## Ethics Statement

None.

## CRediT authorship contribution statement

**András Attila Csontos:** Investigation, Formal analysis, Software, Writing – original draft. **Danijel Šugar:** Investigation, Formal analysis, Project administration, Writing – review & editing.

## Data Availability

DIM_sets (Original data) (Mendeley Data).
